# Effects of a Portable Peritoneal Lavage Device on Dogs with Seawater-Immersed Open Abdominal Injury

**DOI:** 10.1155/2019/6132504

**Published:** 2019-03-26

**Authors:** Song Zhou, Kai Nie, Wenhua Zhang, Zhihong Shao, Wei Zhong, Qinglin Ye, Xiaoqiang Lin, Yuefeng Chen, Xiaowen Wang

**Affiliations:** Department of General Surgery, The Affiliated Southeast Hospital of Xiamen University (the 175th Hospital of PLA), Zhangzhou, China

## Abstract

**Background:**

Seawater-immersed open abdominal injury is a special injury during marine activities. Effective warmed peritoneal lavage in the field early after injury is the key to treatment. This pilot study aimed at exploring the treatment effects of a self-developed portable peritoneal lavage device compared with conventional treatment model.

**Material and Methods:**

Beagle dogs were used to develop models of seawater-immersed open abdominal injury. A conventional lavage method or a novel peritoneal lavage device was used for lavage and rewarming. The vital signs, electrolyte, serum inflammatory cytokine expression levels, histological changes of mucosa, and microstructure variety of different groups were observed and compared before and after immersion and 2 h, 1 d, 3 d, and 5 d after lavage.

**Results:**

The levels of TNF-*α*, IL-1*β*, IL-8, IFN-*γ*, VEGF, and TGF-*β* in the blood and the damage of tissues and cells in three groups were increased after immersion and decreased at the later points of time after lavage. The concentration of Na^+^, K^+^, Cl^−^, lactate, and lactate dehydrogenase in the plasma was significantly higher than that before immersion (*P* < 0.05), and the concentration of Ca^2+^ and HCO_3_^−^ and plasma pH decreased slightly (*P* < 0.05). The degree of tissue inflammation and mucosal injury in the delayed control group and device group was lower than the control group.

**Conclusions:**

Timely lavage and rewarming using a portable peritoneal lavage device reduced the inflammatory response of seawater-immersed open abdominal injury dogs and reduced the damage of multiple organs. The dogs recovered better and faster than the conventional treatment group.

## 1. Introduction

In the recent years, with the development of modern technology and the gradual increase in human exploration and exploitation of marine resources, marine activities have become very common. Naval battle becomes the main form of modern warfare, and many high-tech powerful weapons were used than ever before. Marine operations, dangerous marine life attacks, naval wars, and other accidents lead to the falling of the wounded into the sea and developing seawater-immersed injury [1]. Seawater-immersed open abdominal injury is a special injury during these marine activities. Seawater is a hypertonic liquid with large thermal conductivity and low temperature and contains a variety of pathogenic microorganisms. Therefore, seawater-immersed open injury causes not only direct abdominal injury but also sodium hyperlipidemia and dehydration, resulting in internal environment disorders, severe infections, and multiple organ dysfunction more easily than ordinary open abdominal wounds.

Inflammatory reactions constitute an essential part in a secondary injury, and also, they are the major cause for the progressive aggravation of injury. The second hit can then increase the risk for multiple organ dysfunction syndrome (MODS) and multiple organ failure (MOF) [2]. Studies have shown that early lavage rewarming after seawater immersion injury is of great importance to reduce the incidence of MODS and improve the success rate of treatment [3]. Effective warmed peritoneal lavage in the field early after injury is the key to treatment. To the best of our knowledge, no study was found in the literature to design a peritoneal lavage device and to investigate its effects on seawater-immersed open abdominal injury. Therefore, in this study, a portable peritoneal lavage device developed by ourselves was used to explore the advantages of lavage and rewarming compared with the conventional treatment mode.

## 2. Materials and Methods

### 2.1. Portable Peritoneal Lavage Device

**Figure 1:**
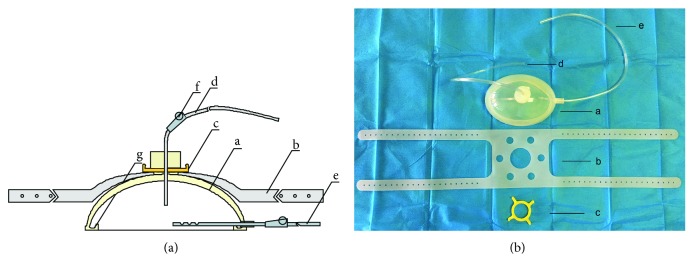
The design and figure for a portable peritoneal lavage device. (a) The design of the portable peritoneal lavage device, and (b) the physical map of the device. It consists of three parts: an abdominal cover (A), a strap (B), and a fixed bracket (C). The abdominal cover is the main part of the device, including the main body of the abdominal cover and the inlet and outlet pipes (D and E).

As previously reported [4], the portable peritoneal lavage device consists of three parts: a celiac cover (A), strap (B), and fixed bracket (C) (Figure 1). The abdominal cover is the main part of the device, including the main body of the abdominal cover and the inlet and outlet pipes (D and E). Each pipe contains a stop valve (F). The cover is thinned from top to bottom and is provided with a convex edge (G). By using the strap and bracket, the cover can be fixed on the abdomen. In addition, the cover can be attracted to the injures' abdomen by a certain deformation through a thinner structure at the bottom end. Considering electric power is often inaccessible outside; no pumps were used in the device. The lavage fluid is forced by gravity and air pressure and flows through the inlet pipe.

### 2.2. Experimental Animals and Study Design

This study was approved by the Animal Experiment Committee of Xiamen University, and all the dogs were handled according to the Declaration of the National Institutes of Health Guide for Care and Use of Laboratory Animals. 20 male beagle dogs (15~18 kg) were selected for the study. The dogs were given standard dog food and free access to water. 15 dogs were randomly divided into three groups (*n* = 5): the control group (group A): abdominal open wound + seawater immersion + conventional delayed lavage; the delayed control group (group B): open abdominal wound + seawater immersion + conventional lavage; the device group (group C): open abdominal wound + seawater immersion + device lavage. Another 5 dogs were prepared for histopathological and ultrastructure examination. To eliminate the influence of biorhythm on the experimental results, all the experiments were started at 8 am.

### 2.3. Preparation of Artificial Seawater

The artificial seawater was prepared according to the data from the Third Institute of Oceanography of the State Oceanic Administration of the People's Republic of China. The main indexes were infiltration concentration 1250 ± 11.52 mmol/L, pH 8.2, sodium ion concentration 630 ± 5.33 mmol/L, potassium ion concentration 10.88 ± 0.68 mmol/L, chloride ion concentration 658.8 ± 5.25 mmol/L, and temperature 21°C. Laboratory temperature was 25°C.

### 2.4. Establishment of Animal Model

Each dog was anesthetized with an intramuscular injection of ketamine (20 mg/kg) and then fixed with supine position on a self-made surgical holder. The dogs were preoperatively catheterized through the femoral artery and femoral vein of the forelimb, and propofol (3 mg/kg/h) was used to maintain anesthesia intraoperatively. Ventral midline incision of 8 cm was performed by scalpel to create a surgical abdominal open injury. The dogs were immersed in artificial seawater at 25°C for 3 h after injury. The immersion surface was over the xiphoid for 5 cm. Three hours later, the dogs were pulled up from seawater. The control group (group A) was used to mimic the present treatment mode. The dogs in this group were placed at room temperature for 3 h, then warmed peritoneal lavage was performed using hypotonic saline chloride solution (0.45%, 40°C). The dogs in group B were treated with hypotonic saline chloride solution immediately after the dogs were pulled up from the seawater. For group C, the dogs were treated with a portable peritoneal lavage device with hypotonic saline chloride solution for lavage and rewarming. The total amount of lavage fluid is 2 L. After surgery, the abdominal injuries were sutured, and the dogs were brought back to the cages individually.

### 2.5. Vital Signs Monitoring

All animals were catheterized from the femoral artery and vein, and pressure transducers were connected to an invasive EKG monitor. The heart rate, mean arterial pressure (MAP) and venous pressure (CVP) were monitored before immersion, after immersion and 2 h after lavage. The temperature was measured at the above time points by inserting the thermometer into the anus.

### 2.6. Serum Inflammatory Cytokines, Electrolytes, and Biochemical Markers

Blood samples were drawn 5 mL before injury, 3 h after injury, and 2 h, 1 day, 3 days, and 5 days after lavage. Serum was collected after centrifugation, and the levels of TNF-*α*, IFN-*γ*, IL-1*β*, IL-6, IL-8, VEGF, TGF-*β*, and endotoxin were determined by enzyme-linked immunosorbent assay (ELISA). Arterial blood gas, electrolytes, lactate, and lactate dehydrogenase were detected by the hospital laboratory.

### 2.7. Histopathological Examination

Another 5 beagle dogs were used for this experiment. The dogs were euthanized at the following time points: 2 h after open abdominal injury, 3 h after the dogs were pulled up from seawater, 2 h after delayed lavage, 2 h after lavage, and 2 h after lavage with the device. The liver, lung, stomach, small intestine, and heart tissues were harvested immediately after the dogs were killed. After neutral formaldehyde fixation, paraffin embedding, sectioning, and HE staining, the pathological changes of the tissues were observed under a microscope.

### 2.8. Transmission Electron Microscope Observation

The liver and small intestinal tissues were fixed for 2 hours in a mixture (pH 7.4) of 2.5% glutaraldehyde and 4% paraformaldehyde in PBS. The tissues were cut to a size of 4 mm in length and 2 mm in width and further embedded and sliced. The ultrastructure of tissues was examined and photographed with transmission electron microscope (JEM2100HC; JEOL, Tokyo, Japan).

### 2.9. Statistical Method

Statistics were analyzed with SPSS 19.0 software (SPSS Inc., Chicago, IL, USA). All data are expressed as mean ± SD. One-way analysis of variance (ANOVA) was used for multiple comparisons. *P* < 0.05 was considered statistically significant.

## 3. Results

### 3.1. Influence of a Portable Lavage Device on the Vital Sign of Dogs with Seawater-Immersed Open Abdominal Injury

**Figure 2:**
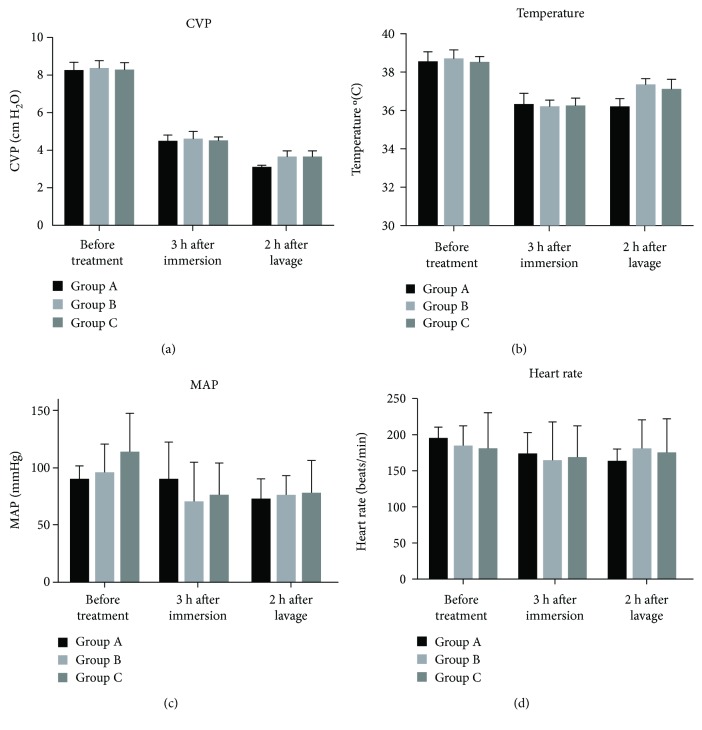
Comparison of the vital signs among different groups. Dogs were treated with delayed lavage (group A), timely lavage (group B), and timely lavage with peritoneal lavage device (group C); the CVP, MAP, temperature, and heart rate were detected at each time point. CVP: central venous pressure; MAP: mean artery pressure.

There was no significant difference in MAP, CVP, body temperature, and heart rate between the three groups before immersion (Figure 2). After the dogs were immersed in seawater for 3 h, the CVP and body temperature decreased significantly (*P* < 0.05). The MAP and heart rate also decreased. The CVP decreased further 2 h after rinsing (*P* < 0.05). The body temperature, MAP, and heart rate in group A continued to decline, while the body temperature, MAP, and heart rate in group B and C increased after lavage.

### 3.2. Effect of a Portable Peritoneal Lavage Device on Inflammatory Cytokines in Dogs with Seawater-Immersed Open Abdominal Injury

The concentrations of endotoxin, TNF-*α*, IL-1*β*, IL-6, IL-8, IFN-*γ*, VEGF, and TGF-*β* at different time points in the three groups of dogs were shown in Table 1. Endotoxin in the blood was rapidly increased after immersion in seawater, and it still showed an upward trend after lavage. IL-1*β*, IL-6, IL-8, VEGF, and GF-*β* reached the peak 1 d after lavage. The expression level of IL-6 and TGF-*β* in group B and C at 2 h after lavage is relatively lower than group A (*P* < 0.05). TNF-*α*, IL-1*β*, IL-8, IFN-*γ*, VEGF, and TGF-*β* in group C were significantly decreased compared with group A (*P* < 0.05). There was no big difference between group B and group C on the expression of cytokines.

### 3.3. Effect of a Portable Peritoneal Lavage Device on Electrolytes, Lactate, and Lactate Dehydrogenase of Dogs with Seawater-Immersed Open Abdominal Injury

The concentration of Na^+^, K^+^, and Cl^−^ in the plasma of the dogs after immersion in seawater was significantly higher than that before immersion (*P* < 0.05), the concentration of HCO_3_^−^ and the plasma pH was slightly decreased. After warmed peritoneal lavage, the concentrations of Na^+^, K^+^, and Cl^−^ decreased gradually (*P* < 0.05) on 1 d, 3 d, and 5 d after immersion and returned to normal on the 5th day after lavage (*P* < 0.05). The concentrations of Na^+^, K^+^, Cl^−^, and pH in the device group after 1 day of lavage were recovered better than those in the control group (*P* < 0.05). HCO3^−^ concentration and pH were adjusted after lavage, which was statistically significant at 3 h after lavage (*P* < 0.05) (Table 2). Lactate acid and lactate dehydrogenase (LDH) concentrations in the blood of dogs were significantly increased 3 h after lavage (*P* < 0.05). Lactic acid concentration decreased, while the concentration of lactate dehydrogenase continued to rise after conventional time-lapse rinse or device rinsing (Table 2).

### 3.4. Effects of a Portable Peritoneal Lavage Device on Organ Inflammation of Dogs with Seawater-Immersed Open Abdominal Injury

**Figure 3:**
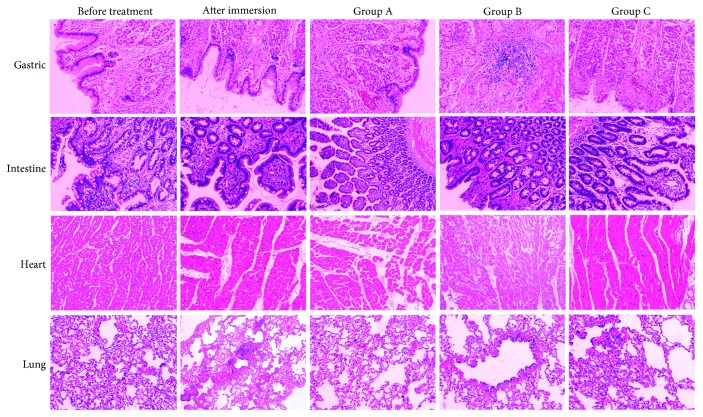
H-E staining of the stomach, small intestine, heart, and lung tissue in dogs with different treatments at each time point (100x). Dogs were euthanized at 2 h after open abdominal injury, 3 h after the dogs were pulled up from seawater, 2 h after delayed lavage (group A), 2 h after timely lavage (group B), and 2 h after timely lavage with the device (group C). The lung, stomach, small intestine, and heart tissues were harvested immediately after the dogs were euthanized. After fixation, paraffin embedding, sectioning, and HE staining, the pathological changes of the tissues were observed under a microscope.

Before exposed to seawater, the dogs had intact small intestine mucosa, clear villi structure, and neat villus arrangement, and no significant hepatic edema, necrosis, and inflammatory cell infiltration in the liver (Figure 3). Seawater immersion caused acute intestinal mucosa injury and inflammation which were represented by massive destruction of villi and inflammatory cell infiltration into the lamina propria. The inflammatory cells in the gastric tissue were also aggregated; the microvilli were shed and ruptured. The gastrointestinal mucosal injury in group B and C was lighter than group A, and mitochondrial swelling was reduced. No significant difference was observed in the heart and lung tissues.

### 3.5. Effects of a Portable Peritoneal Lavage Device on Tissue Ultrastructure of Dogs with Seawater-Immersed Open Abdominal Injury

**Figure 4:**
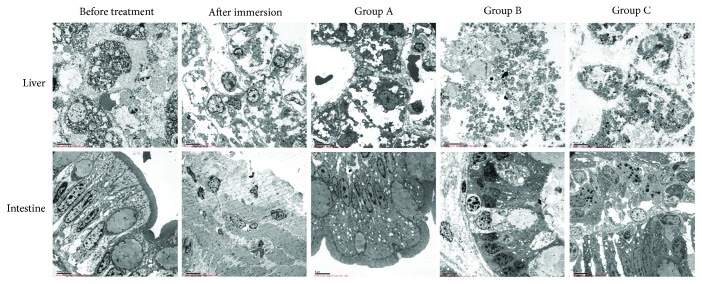
Ultrastructure of the liver, stomach, and small intestine cells in different treatment groups. Dogs were euthanized at 2 h after open abdominal injury, 3 h after the dogs were pulled up from seawater, 2 h after delayed lavage (group A), 2 h after timely lavage (group B), and 2 h after timely lavage with the device (group C). The small intestine and liver tissues were obtained immediately after the dogs were euthanized. After fixation, embedding and sectioning, the ultrastructure changes of the tissues were observed under a microscope (2000x).

The ultrastructure of the liver, stomach, and small intestine in different treatment groups was observed by transmission electron microscopy (Figure 4). Before seawater immersion, the nuclei in liver cells showed an even, round contour, heterochromatin adjacent to their external border and distinct nuclear membranes and nuclear pores. The mitochondria were slightly swollen. Compared to the control group, we observed obvious ultrastructural changes in the liver after open abdomen injury and seawater immersion in the dogs from group A, B, and C. But there was no significant difference between these three groups. We can see swelled mitochondria and incomplete cell membrane; nuclear chromatin and cytoplasm have a certain degree of dissolution, and part of the cells undergo necrosis. Before immersion, intestinal villus was integrated and arranged neatly. After lavage and immersed in seawater, the intestinal epithelial cells were vacuolar degeneration, rough endoplasmic reticulum dilatation, mild mitochondria swelling, part of goblet cells emptying, and interstitial edema with infiltration of inflammatory cells.

## 4. Discussion

Seawater is a kind of high permeability and high alkaline liquid with large thermal conductivity, low temperature, and high bacteria content. Highly permeable alkaline seawater causes an increased local osmolarity of the open abdominal cavity, intracellular dehydration, and tissue space edema. These states directly stimulate histiocytes to release inflammatory mediators such as IL-8, TNF-*α*, and TGF-*β* and aggravate the hemorrhage of small vessels in injured tissues [5]. A large number of pathogenic bacteria and toxins in the seawater can enter the blood circulation through the peritoneum, resulting in systemic diseases such as infection, shock, and MOF [6]. In addition, prolonged seawater immersion causes adverse reactions, including hypothermia, cardiovascular disturbances, heart failure, metabolic acidosis, and renal failure [7].

When a patient suffers of seawater immersion in combination with open abdominal injuries, the first thing we should do is to pull the patient out of the seawater environment. Then measures such as debridement, rewarming, correcting water and electrolyte disorders, anti-infection treatment [8], and sometimes damage control surgery [9, 10] are needed. The only method to treat hypothermia is the rise of the patient's core temperature [11]. Different methods can be used to achieve this goal. However, for patients with hypothermia and open abdominal injury, the simplest and most effective method is peritoneal lavage. Early use of warm hypotonic saline solution for peritoneal lavage reduced the functional damage of low temperature seawater on the viscera and improved the survival rate of seawater-immersed open abdominal injury experimental animals [12, 13]. However, under certain circumstances on the sea, due to the restriction of conditions, there are difficulties in rescuing the wounded. The time window for surgical treatment on the sea is longer than that on land, and most of the ships lack good treatment conditions and convenient tools for evacuation. With the increase of evacuation time, the risk of postoperative complications of primary definitive surgery for abdominal wounds also increased accordingly [14]. The current treatment model for seawater-immersed open abdominal injury patients is wound dressing, rehydration, anti-infective treatment, and then transfer to the nearest hospital. This delayed the time for lavage and rewarming and finally affect the success rate of rescue. Currently, there are no available portable peritoneal lavage device for peritoneal lavage and rewarming.

In this experiment, we designed a portable peritoneal lavage device and established a seawater-immersed open abdominal injury model with dogs. Dogs were treated with three different treatment methods, including peritoneal lavage using a self-developed device. Compared with the control group (simulating the existing delayed lavage rewarming treatment mode), the expression level of TNF-*α*, IL-6, IL-1*β*, IL-8, IFN-*γ*, VEGF, and TGF-*β* was decreased. It also suppressed the degree of inflammation to some extent and reduced the damage of the small intestine, stomach, and liver tissues. That means group B and C had a lower inflammation status compared to group A. In addition, through the lavage and rewarming of hypotonic lavage fluid in a timely manner, the dogs' body temperature recovered; the symptoms of high sodium, potassium, and high chlorine blood disease relieved within a relatively short period of time.

In conclusion, the above results indicate that timely lavage and rewarming using our portable peritoneal lavage device can reduce the inflammatory response of seawater-immersed open abdominal injury dogs and reduce the damage of multiple organs. The comprehensive effects are better than the existing treatment mode, which has an important reference role for the treatment of patients with seawater-immersed open abdominal injury.

## Figures and Tables

**Table 1:** Comparison of the serum inflammation-related indicators of the three groups of dogs (*n* = 5, mean ± SD).

Time	Group	Endotoxin (Eu/L)	TNF-*α* (mg/L)	IL-1*β* (mg/L)	IL-6 (mg/L)	IL-8 (mg/L)	IFN-*γ* (mg/L)	VEGF (mg/L)	TGF-*β* (ng/L)
Before treatment	A	0.19 ± 0.15	18.50 ± 5.62	408.29 ± 76.89^†^	559.39 ± 56.61	126.91 ± 34.99	59.76 ± 10.19	317.14 ± 58.57	70.65 ± 14.13
	B	0.34 ± 0.23	19.07 ± 6.12	383.26 ± 132.61^†^	548.30 ± 179.38	148.28 ± 36.05	62.21 ± 14.70	258.37 ± 48.49	74.28 ± 10.32
	C	0.18 ± 0.09^†^	21.44 ± 7.35	411.55 ± 112.41^†^	558.59 ± 26.46	100.76 ± 22.77	60.30 ± 12.66	249.56 ± 21.66	73.62 ± 12.61

After immersion	A	0.30 ± 0.14	24.94 ± 4.78	646.54 ± 54.12	681.59 ± 105.75	171.50 ± 48.28	73.35 ± 12.67	346.79 ± 114.31	81.69 ± 10.27
	B	0.53 ± 0.21	27.02 ± 5.81	647.16 ± 121.47	573.08 ± 122.22	149.08 ± 46.31	75.66 ± 18.79	297.88 ± 60.82	86.98 ± 17.59
	C	0.50 ± 0.15	26.12 ± 4.09	685.80 ± 89.77	581.76 ± 87.98	120.51 ± 40.56	65.26 ± 11.87	246.99 ± 22.95	84.45 ± 16.59

2 h after lavage	A	1.07 ± 0.26^†^	34.22 ± 4.15^†^	722.35 ± 29.20^†^	805.41 ± 130.60	210.37 ± 48.03	82.47 ± 13.44	325.39 ± 73.89	116.21 ± 20.80^†^
	B	0.89 ± 0.10^†^	27.86 ± 3.28	694.42 ± 75.20	631.61 ± 52.07^∗^	163.48 ± 34.78	76.14 ± 13.70	295.12 ± 48.27	84.27 ± 8.53^∗^
	C	0.69 ± 0.22^∗^	27.85 ± 4.75	636.61 ± 137.16	625.75 ± 84.74^∗^	144.74 ± 37.17^∗^	65.24 ± 15.22	268.30 ± 14.27	89.05 ± 10.55^∗^

1 d after lavage	A	0.73 ± 0.25^†^	33.23 ± 4.47^†^	879.37 ± 40.27^†^	770.07 ± 119.47	237.45 ± 36.24^†^	89.40 ± 9.31	475.16 ± 103.27	126.96 ± 16.83^†^
	B	0.96 ± 0.24^†^	28.74 ± 3.98	766.80 ± 82.71	614.24 ± 21.04^∗^	169.41 ± 36.64^∗^	77.37 ± 11.92	337.94 ± 92.38	96.02 ± 17.87^∗^
	C	0.94 ± 0.30^†^	22.13 ± 8.26^∗^	659.85 ± 119.99^∗^	701.68 ± 77.13	163.42 ± 34.57^∗^	58.36 ± 11.49^∗^	290.21 ± 48.38^∗^	97.92 ± 14.58^∗^

3 d after lavage	A	1.07 ± 0.17^†^	33.71 ± 4.48^†^	759.99 ± 73.71^†^	728.45 ± 117.11	201.21 ± 38.95	83.85 ± 15.16	482.44 ± 110.36	110.76 ± 17.86^†^
	B	0.95 ± 0.15^†^	23.61 ± 2.86^∗^	638.65 ± 46.12	612.98 ± 72.94	184.80 ± 17.09	71.07 ± 4.64	292.67 ± 67.65^∗^	91.07 ± 19.92
	C	0.68 ± 0.21^∗^	24.09 ± 2.56^∗^	622.06 ± 90.91^∗^	631.66 ± 151.79	143.08 ± 57.94	63.99 ± 7.74^∗^	306.73 ± 61.97^∗^	86.80 ± 22.13

5 d after lavage	A	0.88 ± 0.32^†^	25.82 ± 3.29	721.35 ± 83.57	602.35 ± 155.95	202.80 ± 34.90	82.84 ± 11.73	473.84 ± 108.65	125.52 ± 9.49^†^
	B	0.79 ± 0.11^†^	20.76 ± 2.17	614.79 ± 91.31	574.91 ± 57.51	172.03 ± 32.50	60.82 ± 5.80^∗^	310.51 ± 71.01	90.43 ± 26.86
	C	0.56 ± 0.18	23.20 ± 4.89	608.58 ± 81.77	643.49 ± 136.27	163.37 ± 44.84	59.35 ± 5.42^∗^	278.10 ± 49.20^∗^	89.50 ± 18.20

^∗^ *P* < 0.05 compared with group A; ^†^*P* < 0.05 groups at each time point compared with the data at the time after immersion, respectively.

**Table 2:** Detection of electrolyte, blood gas, and biochemical indicators from the two groups of dogs at different time points (*n* = 5, mean ± SD).

Time	Group	Ca^2+^ (mmol/L)	K^+^ (mmol/L)	Na^+^ (mmol/L)	Cl^−^ (mmol/L)	HCO_3_^−^ (mmol/L)	pH	Lactic acid (mmol/L)	LDH (*μ*/L)
Before treatment	A	2.38 ± 0.09	3.58 ± 0.08^†^	142.96 ± 3.23^†^	112.78 ± 2.79^†^	18.24 ± 0.59^†^	7.41 ± 0.02^†^	6.95 ± 0.17^†^	256.06 ± 28.18^†^
	B	2.42 ± 0.12	3.64 ± 0.12^†^	144.24 ± 1.66^†^	112.36 ± 2.23^†^	18.28 ± 0.58^†^	7.41 ± 0.01^†^	6.61 ± 0.44^†^	244.40 ± 23.25^†^
	C	2.42 ± 0.10	3.60 ± 0.14^†^	143.24 ± 2.14^†^	111.32 ± 1.92^†^	18.06 ± 0.87^†^	7.40 ± 0.03^†^	6.55 ± 0.37^†^	238.50 ± 35.30^†^

After immersion	A	2.43 ± 0.13	4.03 ± 0.15	151.02 ± 2.84	127.96 ± 3.14	14.58 ± 0.86	7.29 ± 0.04	8.27 ± 0.33	380.02 ± 45.89
	B	2.46 ± 0.17	4.00 ± 0.18	153.50 ± 3.25	124.82 ± 1.18	14.22 ± 1.13	7.30 ± 0.02	8.16 ± 0.19	359.74 ± 20.42
	C	2.39 ± 0.10	4.12 ± 0.20	153.70 ± 3.84	124.96 ± 2.09	14.66 ± 1.00	7.29 ± 0.05	8.19 ± 0.21	365.94 ± 27.91

2 h after lavage	A	2.38 ± 0.11	4.03 ± 0.12	148.08 ± 1.45	125.34 ± 2.16	14.92 ± 0.75	7.31 ± 0.03	8.17 ± 0.16	615.16 ± 19.88^†^
	B	2.33 ± 0.15	3.88 ± 0.08	146.44 ± 1.80	122.14 ± 3.40	15.54 ± 0.46^†^	7.36 ± 0.02^†^	7.78 ± 0.07^†^	581.98 ± 29.12^†^
	C	2.30 ± 0.09	3.79 ± 0.10^∗†^	144.96 ± 1.59^∗†^	121.40 ± 3.75	16.20 ± 0.68^∗†^	7.36 ± 0.02^∗†^	7.74 ± 0.11^∗†^	593.30 ± 35.29^†^

1 d after lavage	A	2.40 ± 0.09	3.84 ± 0.07^†^	145.60 ± 1.47^†^	122.16 ± 3.39^†^	16.06 ± 0.34^†^	7.33 ± 0.03	7.80 ± 0.31^†^	865.62 ± 49.93^†^
	B	2.41 ± 0.14	3.64 ± 0.20^†^	143.64 ± 1.25^†^	115.88 ± 3.27^†^	17.46 ± 0.93^†^	7.39 ± 0.03^†^	7.06 ± 0.32^†^	732.12 ± 53.10^†^
	C	2.38 ± 0.08	3.54 ± 0.08^∗†^	143.08 ± 0.86^∗†^	117.28 ± 2.32^∗†^	17.36 ± 0.86^†^	7.40 ± 0.03^∗†^	7.08 ± 0.30^∗†^	771.38 ± 33.46^∗†^

3 d after lavage	A	2.36 ± 0.16	3.58 ± 0.10^†^	143.10 ± 0.75^†^	114.72 ± 3.16^†^	17.98 ± 0.50^†^	7.39 ± 0.02^†^	6.86 ± 0.50^†^	1058.78 ± 120.71^†^
	B	2.43 ± 0.12	3.54 ± 0.21^†^	142.12 ± 2.86^†^	111.50 ± 1.08^†^	18.40 ± 0.61^†^	7.40 ± 0.02	6.79 ± 0.37^†^	1008.24 ± 69.31^†^
	C	2.41 ± 0.10	3.44 ± 0.14^†^	141.80 ± 2.32^†^	110.68 ± 2.11^∗†^	18.14 ± 0.42^∗†^	7.40 ± 0.03^†^	6.70 ± 0.38^†^	910.70 ± 65.71^†^

5 d after lavage	A	2.35 ± 0.14	3.47 ± 0.06^†^	141.06 ± 1.01^†^	111.16 ± 1.53^†^	17.68 ± 0.36^†^	7.40 ± 0.02^†^	6.56 ± 0.20^†^	941.84 ± 42.52^†^
	B	2.44 ± 0.07	3.44 ± 0.13^†^	141.26 ± 1.31^†^	110.22 ± 1.66^†^	18.02 ± 0.26^†^	7.40 ± 0.02^†^	6.57 ± 0.27^†^	854.26 ± 63.84^†^
	C	2.41 ± 0.05	3.45 ± 0.07^†^	141.06 ± 1.39^†^	109.82 ± 1.00^†^	18.00 ± 0.29^†^	7.41 ± 0.03^†^	6.64 ± 0.48^†^	931.56 ± 86.64^†^

LDH: lactate dehydrogenase. ^∗^*P* < 0.05 compared with group A; ^†^*P* < 0.05 groups at each time point compared with the data at 3 h after immersion, respectively.

## Data Availability

The data used to support the findings of this study are available from the corresponding author upon request.
